# Reproducibility of native and contrast-enhanced CMR techniques to measure lesion size following acute myocardial infarction

**DOI:** 10.1186/1532-429X-18-S1-P92

**Published:** 2016-01-27

**Authors:** Enver Tahir, Martin R Sinn, Maxim Avanesov, Joshua Wien, Dennis Saering, Christian Stehning, Ulf K Radunski, Kai Muellerleile, Gerhard Adam, Gunnar Lund

**Affiliations:** 1 grid.13648.380000000121803484Diagnostic and Interventional Radiology, University Hospital Eppendorf, Hamburg, Germany; 2 grid.13648.380000000121803484General and Interventional Cardiology, University Medical Center Hamburg-Eppendorf, Hamburg, Germany; 3 Institute of Applied Sciences, Wedel, Germany; 4 Phillips Research Laboratory, Hamburg, Germany

## Background

The purpose of this study was to analyze the reproducibility of native and contrast-enhanced CMR techniques to measure lesion size after acute myocardial infarction (AMI) using native T1/T2 mapping, T2-weighted (T2w) imaging, contrast-enhanced late gadolinium enhancement (LGE), post-contrast T1 mapping and extracellular volume (ECV) quantification.

## Methods

Lesion size was independently quantified by 2 experienced observers in total of 120 consecutive CMRs obtained in 30 patients within the first 6 months after AMI using native and contrast-enhanced sequences. Lesion sizes were quantified using a threshold method (cutoff >2SD of remote normal myocardium) on basal, midventricular and apical short-axis left ventricular slices. Lesion size is given as the mean of both observers. Bland-Altman analysis was performed to determine the agreement between the two observers. Non-parametric Levene's test was used to compare the variances of the relative differences. Statistical analysis was performed using GraphPad Prism 6.

## Results

**Figure 1:**
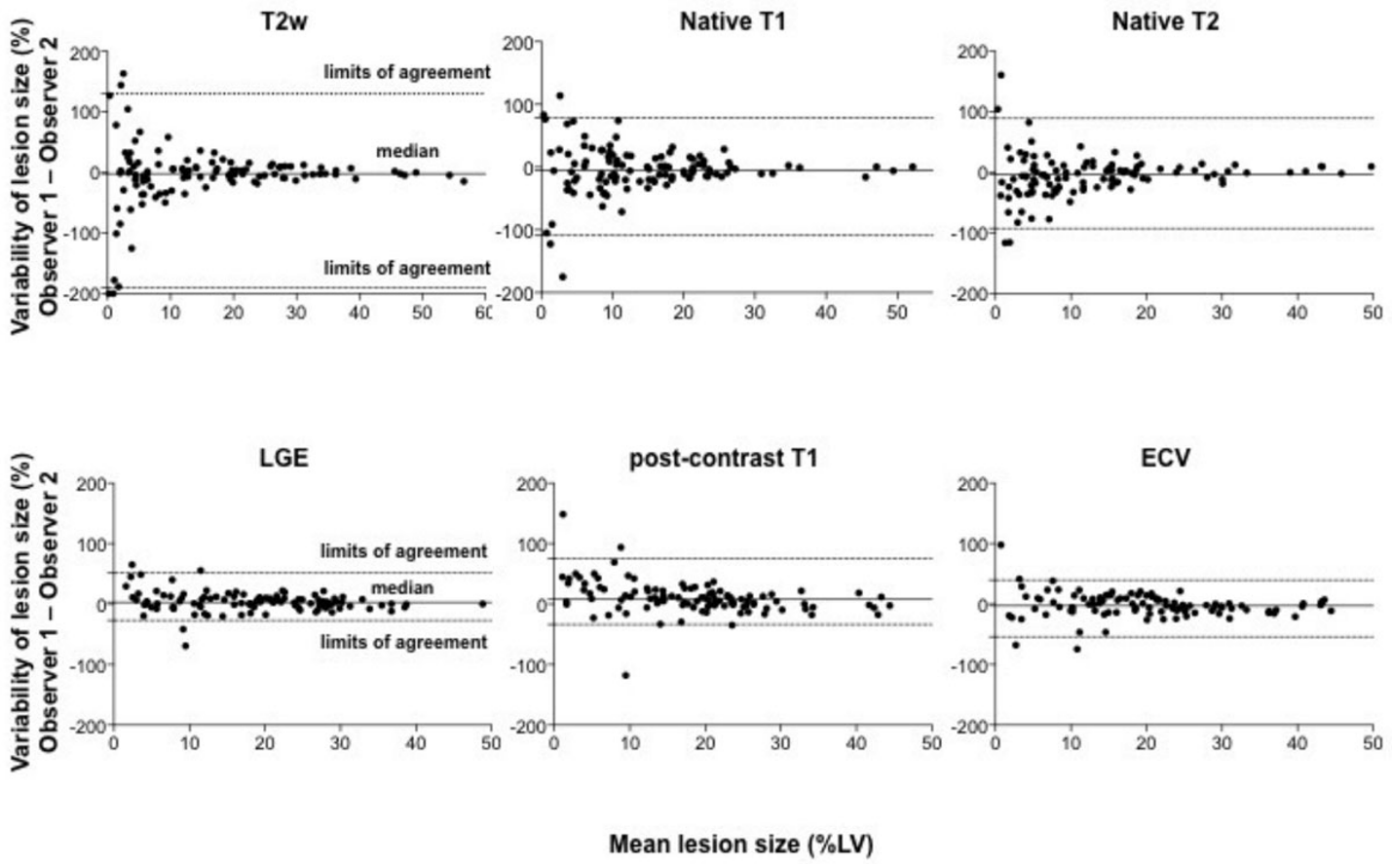
**The Bland-Altman graphs show the relative differences and limits of agreement for measurement of lesion size using the indicated sequences**.

The relative median difference of the native CMR techniques were -1.95% (-12.7% and 9.8%) for T2w, -5.3% (-19.6% and 14.8%) for native T1 and -4.0% (-23.9% and 9.9%) for native T2 (Fig. 1). Results for contrast-enhanced CMR imaging were: 2.9% (-4.5% and 10.5%) for LGE, 7.5% (-2.4% and 21.5%) for post-contrast T1 and -2.9% (-11.4% and 9%) for ECV measurement. Bland Altman analysis revealed a better agreement for all post-contrast sequences indicted by lower limits of agreement compared to native sequences (Figure 1). The increased variability of native imaging was caused by higher interobserver differences in small lesions with sizes between 0-15 %LV compared to lager lesions >15 %LV. This bias was not observed for post-contrast imaging.

## Conclusions

In general, there was a good agreement between the two observers to measure lesion size after AMI, but all post-contrast sequences had a better agreement compared to the native sequences. The low agreement of native imaging was mainly caused by higher interobserver differences in small lesions with lesion sizes between 0-15 %LV compared to lager lesions >15 %LV.

